# Characterization of the “a” determinant region of the hepatitis B virus genome in Iranian patients at different clinical phases of chronic infection 

**Published:** 2018

**Authors:** Sara Romani, Seyed Masoud Hosseini, Seyed Reza Mohebbi, Andre Boonstra, Armin Hosseini Razavi, Afsaneh Sharifian

**Affiliations:** 1 *Department of Microbiology, Faculty of Biological Sciences, Shahid Beheshti University, Tehran, Iran *; 2 *Gastroenterology and Liver Diseases Research Center, Research Institute for Gastroenterology and Liver Diseases, Shahid Beheshti University of Medical Sciences, Tehran, Iran *; 3 *Department of Gastroenterology and Hepatology, Erasmus MC—University Medical Center Rotterdam, Rotterdam, Netherlands *; 4 *Basic and Molecular Epidemiology of Gastrointestinal Disorders Research Center, Research Institute for Gastroenterology and Liver Diseases, Shahid Beheshti University of Medical Science, Tehran, Iran *

**Keywords:** “a” determinant region, Chronic hepatitis B infection, Clinical phases, Amino-acid substitution

## Abstract

**Aim:**

To determine the distribution of important mutations of the “a” determinant region in the HBV genome among patients in different clinical phases of HBV infection.

**Background:**

Variations in Hepatitis B infection not only change the outcome of the disease but also the symptoms from which the chronic HBV patients are suffering.

**Methods:**

We have meticulously selected a total of 40 chronic HBV patients from four different subclasses of chronic HBV clinical phases including immune tolerant (IT), immune active (IA), inactive carrier (IC) and hepatitis B e antigen (HBeAg)-negative (ENEG); 10 samples per each phase. Mutations of the “a” determinant region were identified using PCR-Direct sequencing method.

**Results:**

17 amino-acid substitutions at 12 positions inside the “a” determinant were identified in all forty samples; 3 mutations in the IT group, 6 mutations in the IA phase, 3 mutations in the IC patients and 5 mutations in the ENEG phase. Different substitutions were observed in all four clinical phases. The IA phase was the most variant group with the highest number of amino-acid substitutions.

**Conclusion:**

These results did not reveal a strong pattern to distinguish different clinical phases of Chronic HBV infection, but there are some obvious differences regarding the number and position of mutations between these four clinical phases.

## Introduction

 One of the main aims of the translational research in the infectious diseases era is to elucidate the possible relationships between genetic variations of viral reservoir of HBV patients and the clinical manifestations of their chronic disease. Considering that at least two billion people have been infected with HBV and a 350 million portion are suffering from lifelong problems of chronic HBV infection, it is important to predict clinical demonstrations of an infection, in subgroups of HBV patients.

Chronic HBV infection is a dynamic process. Serological and clinical parameters, such as viral load or hepatic inflammation, may change over time and four clinical phases are distinguished: Immune tolerant (IT), immune active (IA), inactive carrier (IC), and hepatitis B e antigen (HBeAg)-negative (ENEG) (1). 

Mutant HBV strains displaying amino-acid substitutions in the region known as the “a” determinant of HBsAg can modify the antigenicity of the protein and may impair virion secretion and HBsAg formation (2-4). Residues 99 to 169 of Hepatitis B Virus S Antigen (HBsAg) cover the “a” determinant amino-acid region, which is located between amino-acid 124 to 147 and is the neutralizing epitope within the major hydrophilic region (MHR) of the surface gene (5). Such mutants have also been reported in association with clinical features of chronic infection (6), with failures of therapy with specific immunoglobulin in newborns from carrier women and in liver-transplant recipients (7-15) and also escape from vaccine- induced immunity (16-21). For instance, the sD144E/G145R (rtG153E) mutation could lead to antibody-associated escape (22). Similar escape mutations in the “a” determinant could also arise in chronic patients who have not been immunized and result in active viral replication and liver disease after seroconversion from HBsAg to anti- HBs (23, 24). The amino-acid substitution Gly145Arg has been reported most frequently (25), and is known to be stable (26). Altered antigenicity of HBV caused by “a” determinant region mutations has been reported by several studies (5, 27, 28). Analysis of amino-acid positions 112–157 revealed single or multiple substitutions in 39% of Spanish chronic carriers (29). 33 “a” determinant variants (32.7%) of 101 Korean patients were detected, involving a total of 59 amino-acid substitutions at 12 positions within the “a” determinant (30). 

Mutations of “a” determinant region were always studied for diagnostic or prophylactic reasons but their effect on the clinical phases of chronic disease is still unclear. The aim of present study was to determine the distribution of important mutations of the “a” determinant region in the HBV genome among patients in different clinical phases of HBV infection. 

## Methods

**Study population**

During an 8-month period from February to October 2014, forty HBsAg-positive, treatment-naïve subjects who attended the Taleghani Hospital, Tehran, Iran were enrolled in this study. Blood samples were collected from HBV patients with confirmed HBsAg positivity for at least six consecutive months and without co-infection with any blood-borne disease (including HIV, HCV and HDV). According to previous studies which have been conducted by our study group and others, HBV genotype D is the dominant species in Iran (31-33). 

**Case selection, including and excluding criteria**

As reported by the European Association for the Study of Liver (EASL), the following parameters are considered as categorical parameters for HBV clinical phases: serum ALT, HBsAg, HBeAg, anti- HBeAg antibody and HBV viral load. Ten patients with normal ALT (defined as less than 40 IU/L for male and less than 20 IU/L for female) for at least six consecutive months, positive HBeAg, negative anti- HBeAg and a viral load of more than 800,000 IU/mL were allocated to the IT group. Amongst the patients with normal ALT (at least for six consecutive months), ten subjects with undetectable serum HBeAg and positive anti-HBeAg and less than 1500 IU/mL viral load were selected for IC group. For IA group, ten individuals with elevated ALT, negative HBeAg and high viral load but less than 800,000 IU/mL were selected and finally, ten HBeAg negative, HBeAb positive patients with fluctuating ALT and viral load were allocated to ENEG group. 

**Blood biochemistry and serological tests**

5 mL of peripheral blood were collected from each patient, serum was isolated and kept in -20⁰C until the test day. Serum HBsAg and anti-HBe Ab positivity were determined by using Diapro ELISA kits (Diagnostics Bioprobes s.r.l, Italy) and Lyasis 330 autoanalyzer (AMS Diagnostics, FL, USA) was used to evaluate the ALT. 

**HBV viral load test using quantitative real-time PCR**

To evaluate the HBV viral load, first viral DNA was isolated from serum using QIAamp DNA mini kit (Cat#51304, Qiagen, USA) followed by quantitative real-time PCR using ABI7500 real-time PCR system (Applied Biosystems, CA, USA) and HBV quantitative real-time PCR kit from Liferiver biotech Co. (Cat#HD-002-02). 

**PCR amplification and direct sequencing**

Primers used for PCR and direct sequencing were obtained from our previous study (34). All PCR products were purified with ethanol precipitation, shrimp alkaline phosphatase (SAP) (Affymetrix, CA, USA) and sequenced bi-directionally using ABI Prism 3130xl genetic analyzer (Applied Biosystems, CA, USA). 

**Sequencing data analysis and statistical analysis**

To cover the whole “a” determinant region including 303 nucleotides from amino-acid 212 to 312, we have amplified a 600-nucleotide amplicon using a semi-nested PCR. Primers used for the amplification and PCR conditions were according to previous study by Mohebbi et al (35). HBV genome sequence obtained from NCBI website (GenBank) with accession number: AF046996. All sequences were subjected to inspection and matching of forward and reverse strand sequences then alignment with Bioedit software (Ibis Bioscience, CA, USA). 

**Statistical analysis**

Frequency of all detected variants was compared between different clinical groups using Chi square test and fisher exact test where appropriate. P value lower than 0.05 was considered as significant. 

## Results

**Demographic and clinical data**

Patients were selected based on serum ALT, HBsAg, HBeAg, anti-HBeAg antibody and HBV DNA levels as described elsewhere (36). The highest mean ALT levels were observed in the IA group (202.7±198.2). The mean ALT levels among the other three phases ranged between 16 and 122. HBV DNA load in serum samples of these study groups ranged from 300 IU/mL to 56,800,000 IU/mL. The highest mean viral load was observed in the IT group and the lowest was in the IC group. 

**Frequency of naturally occurring variations of the “a” determinant region amongst patients in four clinical phases of HBV**

Analysis of the “a” determinant amino-acid revealed single or multiple substitutions in 13 (32.5%) subjects. A total of 17 amino-acid substitutions at 12 positions inside the “a” determinant was identified in all forty samples; three mutations in the IT group, six mutations in the IA phase, three mutations in the IC patients and five mutations in the ENEG phase (table 1). Serine to Leucine variation in position 143 was the most frequent mutation (identified in 5 out of 40 samples of all four clinical phases) and also observed in three clinical phases IT, IC and ENEG. In the IT phase 3 positions have found variant, in the IA group 6 variant positions, in IC and ENEG groups we have found 2 and 4 variant positions, respectively. Table 1 summarizes all individual mutations identified in the four HBV phases. As can be seen in the table 1, not only more numbers of identified substitutions were found in the IA group, but also the IA phase was the most variant group. 

**Table 1:** Clinical data of the 40 CHB patients in four different clinical phases of infection and the amino-acid mutation patterns in the “a” determinant of HBsAg

Clinical Phase of CHB	Patient code	Age (Year)	Gender (M: Male, F: Female)	AST (IU/L)	ALT (IU/L)	HBV Viral Load (IU/mL)	HBeAg (P: Positive, N: Negative)		A determinant Mutation Number	120P>T	120P>S	127P>L	129Q>N	130G>A	131T>I	134Y>N	134Y>F	143S>L	144D>N	144D>E	145G>A
Immune Tolerant	1	30	F	28	18	56800000		P	0												
	2	26	F	9	10	13100000		P	0												
	3	21	F	15	18	5330000		P	1						*						
	4	37	M	15	18	4800000		P	1							*					
	5	22	M	23	19	3650000		P	0												
	6	34	M	18	21	2400000		P	0												
	7	26	M	17	20	2200000		P	0												
	8	18	F	19	17	960000		P	0												
	9	31	M	12	20	87400		P	1									*			
	10	26	F	11	10	79500		P	0												
Immune Active	11	23	M	32	61	489000		N	0												
	12	26	M	98	299	268000		N	0												
	13	49	M	141	67	147000		N	0												
	14	60	F	34	52	134500		N	0												
	15	41	F	76	84	456000		N	1	*											
	16	48	M	1307	1119	326000		N	1			*									
	17	15	M	40	62	615000		N	0												
	18	50	F	96	98	808000		N	4				*	*	*		*				
	19	42	M	67	125	900000		N	0												
	20	24	F	48	60	840000		N	0												
Inactive Carrier	21	38	M	16	21	603		N	0												
	22	57	M	12	14	390		N	1										*		
	23	24	F	14	15	700		N	0												
	24	43	M	14	16	450		N	0												
	25	40	F	14	16	450		N	1									*			
	26	59	F	15	17	300		N	0												
	27	46	F	11	13	1025		N	0												
	28	35	M	19	21	1300		N	0												
	29	46	M	15	17	1000		N	1									*			
	30	31	F	20	17	800		N	0												
ENEG	31	58	F	12	14	3000		N	1											*	
	32	57	F	14	16	14000		N	0												
	33	54	F	12	9	22900		N	1		*										
	34	49	F	21	15	3750		N	2									*			*
	35	47	M	21	25	5100		N	1									*			
	36	45	F	22	23	25000		N	0												
	37	33	F	9	10	2800		N	0												
	38	32	F	26	45	40000		N	0												
	39	30	F	17	21	27000		N	0												
	40	28	M	22	46	326000		N	0												

**Variants displaying one or more amino-acid substitution in the “a” determinant region**

Classification of patients per number of “a” determinant mutations resulted in 3 groups, including patients with 1, 2 or 4 mutations in this specific region. Eleven patients were carrying only 1 mutation and only one individual were reported with 2 and 4 mutations. No amino-acid substitution has been observed in twenty-seven out of forty patients. 

**Figure 1:**
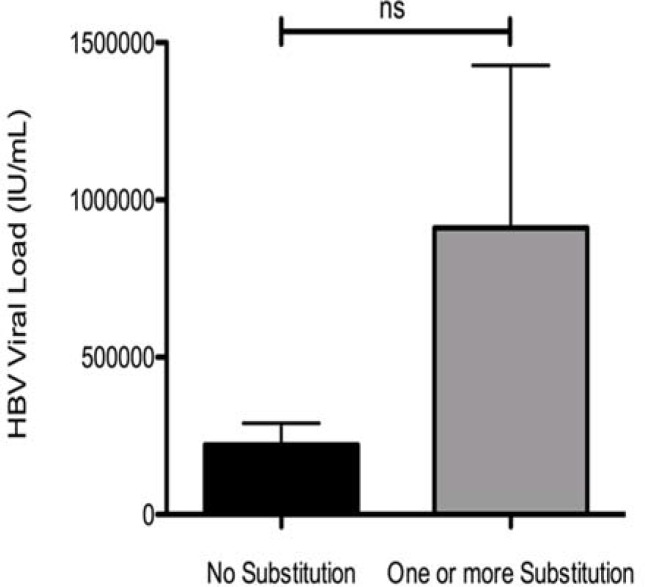
Classification of chronic HBV patients per number of “a” determinant mutations and comparison of HBV viral load between individuals without any substitution and individuals carrying one or more substitution

The mean viral load in carriers of only one mutation was 4 times higher than patients without any mutation and those who have one or more than one mutation (two or three substitutions), 9.1x10^5^ compared to 2.2x10^5^ IU/mL (Figure 1).

## Discussion

The incidence of different clinical outcomes of HBV infection is highly related to the severity and type of immune response to the virus. So, it is assumed that viral genome variants could affect not only the immune response but also the disease outcome in patients carrying genetically different HBV variants. The relationship between “a” determinant region mutations and clinical outcome including clinical phase of chronic disease, vaccine escape and resistance to immunotherapy have not been clearly known so far. Recently, it has been shown that the presence of S mutations was significantly correlated with low HBV DNA and also a high prevalence of naturally occurring HBsAg variants was observed among Tunisian HBV carriers (37). We have selected clinically and laboratory controlled groups of four different clinical phases of HBV infection, and analyzed their “a” determinant region. 

In a nationwide study among Iranian blood donors, 32.8% of the studied strains harbored 195 single or multiple mutations in the MHR (38). Compared to previous results obtained in Japanese, Korean with genotype C (30, 39) and Spanish patients with different genotypes including A, C, D and E (29), a characteristic feature concerning the prevalence of “a” determinant substitutions in our Iranian patients was observed. An unexpectedly higher prevalence of naturally occurring “a” determinant variants (38/40, 95%) was observed in the Iranian patients, compared to 24%, 33% and 39% prevalence in Japanese, Korean and Spanish patients respectively. The high prevalence of “a” determinant variants in Iranian subjects could be the result of the careful selection of patients to classify them into four distinguished clinical phases of CHB in our study compared to other studies, and also genotype D strain features, which could influence biological aspects of the HBV populations in the region, such as rare recombination between genotypes (40).

Ying Shi et al. investigated the prevalence of major hydrophilic region (MHR) mutations, which covers the “a” determinant region, in patients with HBV genotype C, and reported no significant clinical difference between patients with or without MHR mutations (41). In the present study, we also could not find significant correlations between clinical phases and specific mutations. Despite common mutations have been shown in all four clinical phases, each phase (at least at the nucleotide mutations level) had its own mutation that was not observed in other phases (table 1).

Point mutation in residue 145 of S antigen changes the binding capacity of S antigen and is a stable variation and considered as a vaccine escape mutation (42). Mutations of amino-acid 143, 144 and 145 have been found in relation to fulminant reactivation. Our results also showed that 4 out of 7 mutations (58%) found in these three locations were in the reactive phase. In previous studies on changes in G145 locus two more mutations have been found including at the 122 and 123 loci (29), but in our patients we did not find these variations. The position 122 mutation has been reported in a case of a patient with agammaglobulinemia (43). D144N and S143L were also present among two out of ten IC patients in our study population. Co-existence of these two mutations was previously reported as a risk factor for reactivation only in patients with pure mutations not in individuals carrying a mixture of HBsAg variations (44). 

There are several other mutations classified as vaccine escape mutations including T126A, Q129H/R, M133L. We found the concurrent existence of position 129 and 130 mutations in one of the IA patients with positive HBeAb and negative HBeAg. Shahmoradi et al. have identified Q129H mutation in 1.3% of their patients who were HBeAg negative and HBeAb positive (45). Avellon et al. have also found these mutations in one of their patients, with low positive HBsAg tested with 3 different methods. Low antigenicity and activity of the immune system against virus could lead to necroinflammation and progression of fibrosis Mohebbi et al. have identified “a” determinant mutations at amino-acid position 120, 123, 126 and 145. Consistent with our results, they only found G145R in one of their patients (35). In a nationwide study, they also found that the most frequent mutation in MHR of HBV surface antigen (HBsAg) was sP120S (33).

The mean viral load in patients with different numbers of “a” determinant mutations showed a huge difference and this could be due to the nature of those mutations or the effect of their accumulation. It is relevant to mention that a significant effect of pre-core and core promoter region mutations on HBV viral load was reported in Iranian patients (46). 

Our results suggest that “a” determinant mutations should be considered for HBV antigenicity studies, but also when studying the clinical phase of chronic HBV infections. It is still unclear if the mutations, which we studied here, are related to other HBV genome variations or they are useful markers for determining the clinical phase of the infection.
